# Adverse Histopathological Features in Colorectal Cancer Associated with KRAS rs61764370 SNP: A Preliminary Study

**DOI:** 10.3390/biomedicines14020319

**Published:** 2026-01-30

**Authors:** Tradian Ciprian Berisha, Mihai Gabriel Cucu, Alexandru Calotă-Dobrescu, Simona Serban Sosoi, Ana-Maria Ciurea, Alina Maria Mehedințeanu, Puiu Olivian Stovicek, Ramona Adriana Schenker, Cecil Sorin Mirea, Monica-Laura Cara, Florin Burada, Michael Schenker

**Affiliations:** 1 Doctoral School, University of Medicine and Pharmacy Craiova, 200349 Craiova, Romania; berishaciprian@gmail.com; 2 Sf. Nectarie Oncology Center, 200347 Craiova, Romania; amciurea14@gmail.com (A.-M.C.); alina.maria591@gmail.com (A.M.M.); puiuolivian@yahoo.com (P.O.S.); ramona_schenker@yahoo.com (R.A.S.); mike_schenker@yahoo.com (M.S.); 3 Human Genomics Laboratory, University of Medicine and Pharmacy of Craiova, 200349 Craiova, Romania; mihai.cucu@umfcv.ro (M.G.C.); simona.sosoi@umfcv.ro (S.S.S.); 4 Regional Centre of Medical Genetics Dolj, Emergency Clinical County Hospital Craiova, 200642 Craiova, Romania; alexcalota.crgm@gmail.com; 5 Department of Oncology, Faculty of Medicine, University of Medicine and Pharmacy of Craiova, 200349 Craiova, Romania; 6 Department of Pharmacology, Faculty of Nursing, Târgu Jiu Subsidiary, “Titu Maiorescu” University, 040441 Bucharest, Romania; 7 Department of Surgical Semiology, University of Medicine and Pharmacy of Craiova, 200349 Craiova, Romania; cecil.mirea@umfcv.ro; 8 Department of Public Health, Faculty of Medicine, University of Medicine and Pharmacy of Craiova, 200349 Craiova, Romania

**Keywords:** colorectal cancer, *KRAS*, rs61764370, LCS6, SNP, microRNA, aggressiveness, biomarker

## Abstract

**Background/Objectives**: The *KRAS* rs61764370 T>G single-nucleotide polymorphism (SNP), located in a let-7 microRNA binding site within the 3′ untranslated region (3′UTR) of the *KRAS* gene, may modulate tumor aggressiveness by altering post-transcriptional gene regulation. This study evaluated its association with adverse histopathological features in colorectal cancer (CRC). **Methods**: A preliminary study on 83 CRC patients carrying either the TT (wild-type, n = 64) or TG (heterozygous, n = 19) genotype was analyzed. Clinicopathological variables included patient sex, tumor location, American Joint Committee on Cancer (AJCC) staging system, histological grade, perineural invasion (PNI), and lymphovascular invasion (LVI). A composite “tumor aggressiveness” score was defined based on the presence of Grade 3 differentiation, LVI, and/or PNI. Group comparisons were performed using the Chi-square test or Fisher’s exact test, as appropriate. **Results**: No statistically significant differences were observed in sex (*p* = 0.689), tumor location (*p* = 0.781), or stage at diagnosis (*p* = 0.812). Poorly differentiated tumors (Grade 3) were present in 20.3% of TT patients and absent in TG carriers (*p* = 0.06), while low-grade tumors (Grade 1) were more prevalent among TG patients (47.4%) compared to TT (29.7%). The composite high-aggressiveness score was lower in TG (36.8%) than in TT (48.4%), while co-occurrence of PNI and LVI was similar in both groups (~26%). **Conclusions**: Although no significant associations were identified, TG carriers showed a tendency toward lower-grade, less aggressive tumors. Given the limited sample size, these findings should be interpreted with caution, necessitating larger cohorts in order to validate results.

## 1. Introduction

Colorectal cancer (CRC) is one of the most frequently diagnosed malignancies worldwide, representing a significant global health burden [1,2]. According to GLOBOCAN 2022 data, CRC ranks third in incidence and second in cancer-related mortality globally, with over 1.9 million new cases and approximately 935,000 deaths estimated in 2020 alone [3,4]. The epidemiological burden remains substantial in Eastern Europe, where Romania reports one of the highest mortality rates among EU countries [4].

Although the TNM staging system remains the cornerstone of prognostication in CRC, numerous histopathological features provide valuable additional prognostic information. These include tumor grade, mucinous or signet-ring cell histology, lymphovascular invasion (LVI), perineural invasion (PNI), tumor budding, and the presence of tumor deposits [5,6,7,8,9,10,11]. Poorly differentiated tumors, classified by low glandular formation (<50%), have been consistently associated with unfavorable outcomes and higher metastatic potential [5]. Mucinous adenocarcinomas and signet-ring cell carcinomas, considered high-grade subtypes, are known for their aggressive behavior and chemoresistance [6].

Lymphovascular invasion, the presence of tumor cells in endothelial-lined lymphatic or blood vessels, represents a key step in metastatic dissemination and correlates with poor prognosis, particularly in stage II disease [7,9,11]. Perineural invasion, characterized by cancer cell infiltration around or through nerves, is also recognized as an independent predictor of local recurrence, distant metastases, and decreased survival [8,9,10]. Additionally, tumor budding—defined as the presence of isolated single cells or small clusters (<5 cells) at the invasive front—has emerged as an important marker of aggressive biological behavior and adverse outcomes [12].

In recent years, efforts have been made to identify molecular markers that could refine prognostic stratification beyond classical histopathology. Among them, the germline single-nucleotide polymorphism rs61764370, located in the 3′ untranslated region (3′UTR) of the *KRAS* oncogene at the let-7 microRNA binding site (LCS6), has attracted attention [13,14,15,16,17]. The presence of the G allele disrupts binding of the tumor-suppressive let-7 family of microRNAs, potentially leading to increased *KRAS* expression and downstream activation of RAS-MAPK signaling [18].

The let-7 family of microRNAs was originally identified in Caenorhabditis elegans as a key regulator of developmental timing and represents one of the first microRNA families shown to be evolutionarily conserved across species [19,20]. In humans, let-7 microRNAs (hsa-let-7a–h) act predominantly as tumor suppressors by regulating the expression of genes involved in cell cycle progression, cellular differentiation, and apoptosis. Dysregulation and reduced expression of let-7 family members have been reported in multiple malignancies, including colorectal cancer, and have been associated with aggressive tumor behavior and unfavorable clinical outcomes [21,22].

Let-7 microRNAs exert their post-transcriptional regulatory function by binding to complementary sequences within the 3′ untranslated regions (3′UTRs) of target messenger RNAs, leading to translational repression or mRNA destabilization. The *KRAS* oncogene contains a conserved let-7 complementary site (LCS6) within its 3′UTR [23,24]. A single-nucleotide polymorphism, rs61764370 (T>G), located within this region, has been shown to disrupt let-7 binding, resulting in altered *KRAS* regulation and increased oncogenic signaling. In addition to let-7, other microRNAs, including miR-98, have also been reported to target the LCS6 sequence, underscoring the functional relevance of this regulatory region in *KRAS*-mediated tumorigenesis [25,26].

Initial studies linked this polymorphism with poor prognosis in several cancers, including ovarian and lung cancer [18,27], and raised interest in its possible predictive role in colorectal cancer. However, the results have been contradictory. Some studies suggested that the variant G allele may be associated with improved response to anti-EGFR therapy in metastatic CRC [27], while others found no significant correlation with treatment response or overall prognosis [28,29]. Meta-analyses have also failed to confirm a consistent association between rs61764370 and survival outcomes in CRC, highlighting the need for further exploration [30,31].

Given the biological plausibility and its potential regulatory effect on *KRAS* expression, this study aims to explore possible correlations between the rs61764370 SNP and adverse histopathological features in a cohort of colorectal cancer patients. Specifically, the study investigates whether the presence of the G allele correlates with poor differentiation, LVI, PNI, and tumor budding, with the goal of assessing its potential prognostic relevance in clinical practice.

## 2. Materials and Methods

This preliminary analysis includes 83 patients referred for rs61764370 genotyping at the Human Genomics Laboratory of the University of Medicine and Pharmacy in Craiova, Romania, all with histologically confirmed colorectal adenocarcinoma.

The clinical and histopathological variables collected were patient sex, tumor location (colon or rectum), stage at diagnosis (I–IV), histological grade (1–3), presence of perineural invasion (PNI), and lymphovascular invasion (LVI) (Table 1).

To capture the biological complexity of tumor behavior, we introduced a composite aggressiveness score, defined as the presence of at least one of the following adverse histopathological features: poor differentiation (Grade 3), lymphovascular invasion (LVI), or perineural invasion (PNI), and dichotomized tumors as low-grade (Grade 1) versus high-grade (Grades 2 or 3).

### 2.1. SNP Genotyping Analysis

Genomic DNA was extracted from peripheral blood samples using the Wizard^®^ Genomic DNA Purification Kit (Promega, Madison, WI, USA) following the manufacturer’s protocol. DNA concentration and purity were assessed spectrophotometrically; all samples were diluted to a working concentration of 10–20 ng/μL and the target for purity was at least a 1.7 absorption rate at 260 and 320 nm. The single-nucleotide polymorphism rs61764370 in the 3′ untranslated region (3′UTR) of the *KRAS* gene was genotyped using the TaqMan^®^ SNP Genotyping Assay (Applied Biosystems, Waltham, MA, USA), Assay ID: C_89129087_10), which discriminates between the wild-type T allele and the variant G allele. Each reaction was performed in a final volume of 5 μL, containing the following: 2.5 μL of TaqMan^®^ Genotyping Master Mix (Applied Biosystems), 0.25 μL of 20× SNP Genotyping Assay Mix (primers and VIC/FAM-labeled probes), 1.25 μL of genomic DNA (10–20 ng), 1 μL of nuclease-free water.

Reactions were carried out in 384-well optical plates using a ViiA7™ Real-Time PCR System (Thermo Fisher Scientific, Waltham, MA, USA). Thermal cycling conditions consisted of an initial denaturation at 95 °C for 10 min, followed by 40 cycles at 95 °C for 15 s and 60 °C for 1 min, with fluorescence acquisition during annealing/extension; genotypes were automatically determined by allelic discrimination using QuantStudio Software v1.3 based on fluorescence intensity.

All reactions were run in duplicate to ensure reproducibility. Negative controls (no template controls, NTCs) were included in each run to detect potential contamination.

### 2.2. Statistical Analysis

Statistical analyses included descriptive summaries (counts and percentages) and inferential comparisons using Pearson’s chi-square test or Fisher’s exact test, as appropriate. All analyses were performed using IBM SPSS Statistics version 22, with statistical significance set at *p* < 0.05.

## 3. Results

The study cohort consisted of 83 patients diagnosed with colorectal adenocarcinoma, all of them with identifiable *KRAS* rs61764370 polymorphism status.

### 3.1. Demographic and Clinical Characteristics of the Study Population

The sex distribution showed a slight male predominance, with 47 men (56.6%) and 36 women (43.4%).

The youngest patient was 45 years old, while the oldest was 87 years old at the time of diagnosis—a range consistent with the epidemiological profile of colorectal cancer, which predominantly affects older adults. The mean age at diagnosis was 68.6 years.

Tumor localization was almost equally distributed, with 46 cases (55.4%) originating in the colon and 37 cases (44.6%) in the rectum.

Regarding disease stage at diagnosis, most patients were diagnosed with locally advanced or metastatic disease. The most prevalent stage at diagnosis was Stage III (n = 39, 47.0%), followed by Stage IV (n = 20, 24.1%). Earlier stages were less common: twenty-one patients (25.3%) were diagnosed in Stage II and only three patients (3.6%) in Stage I.

Tumor differentiation was predominantly moderate. Grade 2 tumors were identified in 42 patients (50.6%), while 28 patients (33.7%) had well-differentiated (Grade 1) tumors. Poorly differentiated tumors (Grade 3) were found in 13 cases (15.7%).

Invasion-related features were frequent. Perineural invasion (PNI) was present in 39 patients (47.0%) and absent in 44 (53.0%), while lymphovascular invasion (LVI) was present in 35 patients (42.2%) and absent in 48 (57.8%). Co-occurrence of both PNI and LVI was observed in 22 cases (26.5%).

These data reflect a clinically heterogeneous CRC population, representative of real-world diagnostic distributions and staging, with a predominance of advanced-stage and moderately differentiated tumors.

**Figure 1:**
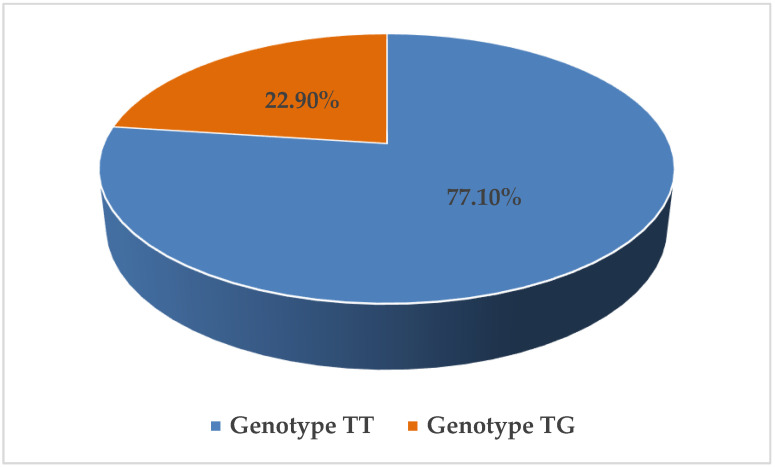
Distribution of TT and TG genotypes in the CRC patient population.

Genetic analysis revealed that 64 patients (77.1%) were homozygous for the wild-type TT genotype, while 19 patients (22.9%) were heterozygous TG carriers of the rs61764370 SNP. (Figure 1).

### 3.2. Demographic and Clinical Characteristics of rs61764370 SNP Subgroups

Demographic characteristics were balanced between the rs61764370 SNP subgroups.

In the TT group, thirty-seven of sixty-four patients (57.8%) were male and there were twenty-seven (42.2%) females, compared to ten males (52.6%) and nine females (47.4%) out of nineteen patients in the TG group. No significant difference in sex distribution was observed (*p* = 0.689).

Similarly, tumor localization was comparable between genotype groups: 36 of 64 TT patients (56.3%) had tumors located in the colon and 28 (43.8%) in the rectum, while among the nineteen heterozygote TG patients, ten (52.6%) had colonic tumors and nine (47.4%) had rectal tumors (*p* = 0.781).

**Figure 2:**
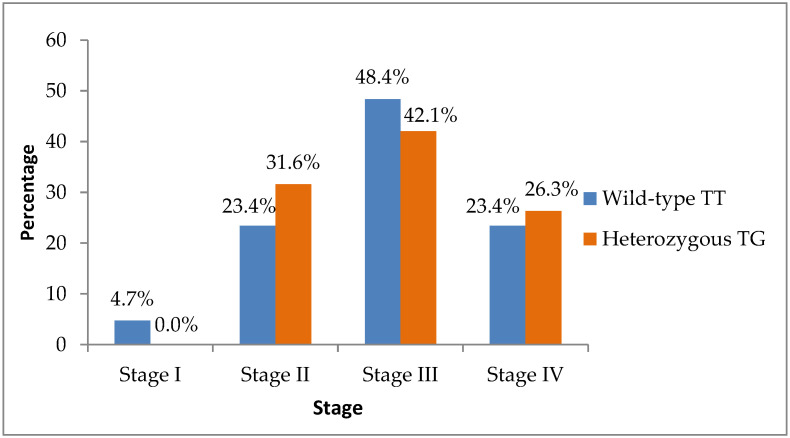
Distribution of tumor stage at diagnosis according to TT (wild-type) and TG (heterozygous) genotypes.

Regarding stage at diagnosis, early-stage disease (AJCC Stage I–II) was present in eighteen of sixty-four TT patients (28.1%) and six of nineteen TG patients (31.6%), whereas advanced-stage disease (Stage III–IV) was observed in forty-six TT (71.9%) and thirteen TG patients (68.4%), relative to their respective subgroup totals. The distribution of disease stage did not differ significantly between groups (Fisher’s exact test, *p* = 0.812) (Figure 2).

Histopathologically, a notable finding was that poorly differentiated tumors (Grade 3) were present in 13 of 64 TT patients (20.3%) but absent in the 19 TG group altogether (0%), which is a difference that approached statistical significance (Fisher’s exact test, *p* = 0.06) (Table 2).

**Figure 3:**
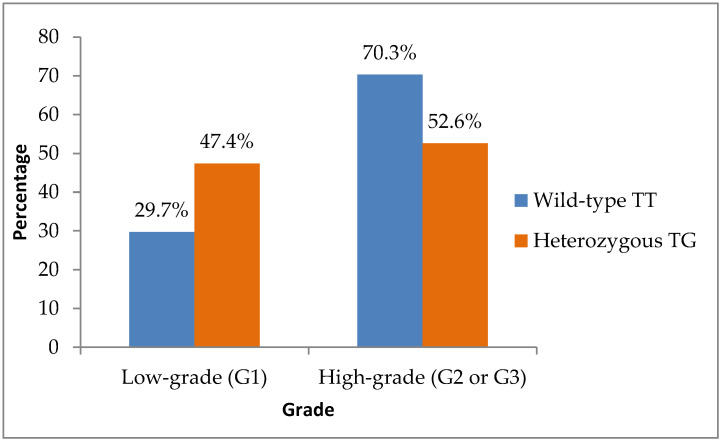
Distribution of tumor grade according to TT (wild-type) and TG (heterozygous) genotypes.

When tumor grade was dichotomized into low-grade (G1) and high-grade (G2 or G3), low-grade tumors (Grade 1) were more frequent among TG patients—accounting for nine of nineteen cases (47.4%), compared with nineteen of sixty-four cases (29.7%) in the TT group. Although this difference did not reach significance (*p* = 0.152), it supported the observed trend toward more favorable histological differentiation in TG carriers (Figure 3).

**Figure 4:**
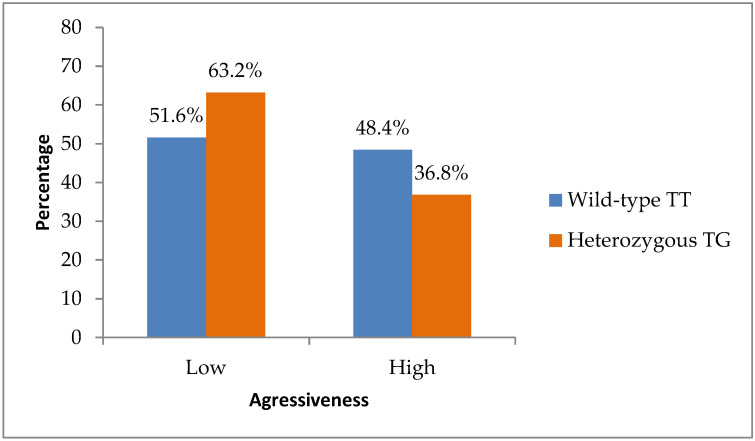
Distribution of tumor aggressiveness to TT (wild-type) and TG (heterozygous) genotypes.

Analysis of a composite tumor aggressiveness score, defined by the presence of any of the following: Grade 3 differentiation, perineural invasion (PNI), or lymphovascular invasion (LVI) revealed a lower proportion of high-aggressiveness tumors among TG patients (seven of nineteen; 36.8%) compared to TT patients (thirty-one of sixty-four; 48.4%). Although this difference was not statistically significant (*p* = 0.422), it aligned with the overall trend, suggesting a less aggressive tumor phenotype in TG carriers (Figure 4).

**Figure 5:**
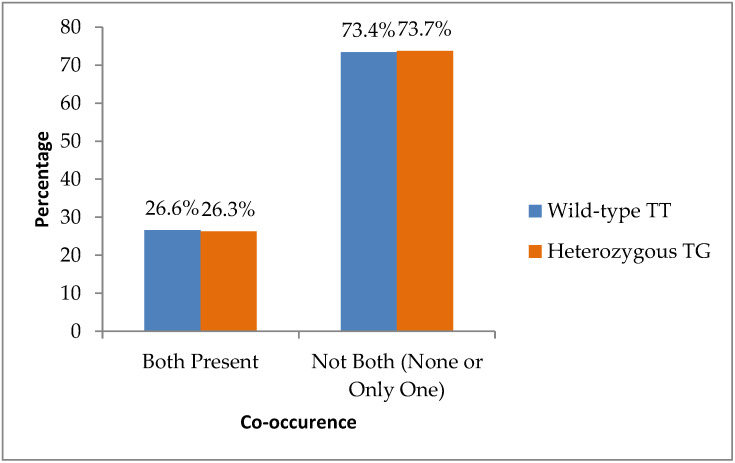
Distribution of co-occurrence of both PNI and LVI status to TT (wild-type) and TG (heterozygous) genotypes.

Finally, co-occurrence of both PNI and LVI was almost identical in the two groups: seventeen of sixty-four TT patients (26.6%) and five of nineteen TG patients (26.3%) (*p* = 0.983) (Figure 5).

While none of these comparisons achieved statistical significance, the patterns suggest a more favorable histopathological profile in TG carriers.

## 4. Discussion

Although no statistically significant associations were observed in this pilot analysis, rs61764370 SNP seems to show a trend toward less aggressive tumor behavior in carriers of the variant genotype (TG). In our study population, none of the patients with the TG genotype had poorly differentiated tumors, and more frequently, they were classified as Grade 1. Moreover, in the analysis based on the composite score, the number of patients with “high-aggressiveness” tumors (presence of one or more features: G3, LVI, and/or PNI) was lower in the TG group compared to the TT group. It is important to note that different histological subtypes carry different grades of aggressiveness [32]. Consequently, we purposefully excluded heterogeneous subtypes by selecting a cohort that consisted solely of adenocarcinoma, the most common form.

The central prognostic importance of histopathological parameters such as tumor differentiation, lymphovascular invasion, and perineural invasion is well-established in colorectal cancer and may, in practice, outweigh the impact of individual germline molecular variants. This concept is supported by recent data from a retrospective cohort of colorectal cancer patients treated with reduced-dose 5-fluorouracil, in which tumor differentiation grade and invasion-related features emerged as the strongest independent predictors of overall survival, whereas *KRAS* mutation status did not demonstrate independent prognostic significance [33].

These findings reinforce the biological and clinical relevance of the histopathological features selected for the composite aggressiveness score in the present study and suggest that subtle germline regulatory variants, such as rs61764370, may exert modulatory rather than dominant prognostic effects.

In terms of underlying mechanisms, the G allele disrupts let-7 miRNA binding within the *KRAS* 3′UTR, potentially resulting in increased *KRAS* expression. However, paradoxically, our results suggest a more favorable histopathological profile in G allele carriers, which implies that the downstream effects of this variant may be context-dependent or influenced by other molecular pathways.

The apparent paradox observed in our cohort—where carriers of the G allele showed trends toward more favorable histopathological features despite the predicted increase in *KRAS* expression—may reflect the complex, context-dependent nature of microRNA-mediated regulation in colorectal cancer. Recent tissue-based analyses of miRNA expression profiles in colon carcinoma have demonstrated that tumor–normal differences in key microRNAs are robust, whereas associations with tumor stage, grade, lymphovascular invasion, or perineural invasion are weak or absent [34]. Notably, tumor-suppressive microRNAs such as miR-185 and miR-141, as well as the oncogenic miR-21, exhibited limited correlation with classical markers of aggressiveness, underscoring that miRNA-driven regulatory effects may influence tumor biology without translating directly into conventional histopathological phenotypes.

Our findings align with those of Smits et al., who reported no statistically significant link between rs61764370 and CRC outcomes, though early-stage disease trends were observed in G allele carriers [29].

Similarly, Kjersem et al. found no predictive impact of the variant in cetuximab-treated patients [28], while Langevin et al.’s meta-analysis concluded that evidence for prognostic relevance remains inconsistent [31].

In contrast, Saridaki et al. noted improved outcomes in G allele carriers treated with anti-EGFR therapy, suggesting potential predictive rather than prognostic roles in specific therapeutic contexts [27].

Beyond colorectal cancer, Ratner et al. demonstrated that the *KRAS* variant is linked to poorer outcomes and platinum resistance in ovarian cancer, which underscores its broader potential as a biomarker of aggressive behavior in certain tumor types [18].

Importantly, most previous studies focused on treatment response or survival endpoints, whereas our analysis explores correlations with established adverse histological features—a novel perspective that may add value to clinicopathological risk stratification.

Furthermore, considering the recent focus on quality of life [35,36,37] in oncological patients, it is important to mention that a recent prospective study by Schenker et al. (2025) [38] evaluated quality-of-life outcomes in rectal cancer patients undergoing chemoradiotherapy, highlighting significant functional decline—particularly in emotional and gastrointestinal domains, most notably among those who required a stoma. This is clinically relevant, as adverse histopathological features such as high-grade differentiation, perineural invasion, and lymphovascular invasion often inform surgical decisions, including stoma formation. Given that the TG genotype group in our study exhibited fewer of these high-risk pathological features, it is plausible that these patients may have had a lower likelihood of requiring a stoma, which in turn could contribute to improved post-treatment quality of life. These findings suggest a potential link between germline variation, tumor biology, surgical management, and patient-centered outcomes.

The present study has several limitations, most notably the relatively small sample size. In addition, the low frequency of the TG genotype, the single-center design, and the absence of multivariate modeling that includes other potentially confounding or effect-modifying molecular markers (e.g., *KRAS* mutation status, MSI, and *BRAF*) should be acknowledged [39].

Nonetheless, the integration of histology and germline variation remains underexplored in CRC.

Despite the extensive investigation of miRNA polymorphisms in colorectal cancer, our study was specifically designed to explore regional variations by comparing cohorts from different ancestral backgrounds. For instance, the rs895819 variant in pre-miR 27a has been associated with increased colorectal cancer susceptibility in Han Chinese populations, with the GG genotype linked to elevated risk and progression [40,41]. Conversely, studies conducted in European cohorts have found no significant association between miR 27a (rs895819), miR 146a, miR 196a 2, miR 492, or miR 608 polymorphisms and colorectal cancer risk [42,43]. Additionally, other research in Chinese populations has reinforced the significant link between rs895819 and colorectal cancer susceptibility [44,45]. These divergent findings between Chinese and European populations underscore the necessity of conducting region-specific analyses. Our cohort, therefore, offers a valuable opportunity to identify population-specific genetic risk factors and deepen our understanding of how regional genetic backgrounds may influence the role of miRNA polymorphisms in colorectal cancer.

Collectively, these observations are consistent with emerging evidence, suggesting that molecular and epigenetic biomarkers in colorectal cancer frequently demonstrate stronger diagnostic or biological relevance than direct prognostic value. Large-scale analyses of miRNA expression profiles have shown clear tumor–normal discrimination but minimal association with survival or invasion-related endpoints [34], while clinical outcome studies continue to identify histological grade and invasion patterns as the primary determinants of prognosis irrespective of *KRAS* mutational status [33]. Within this framework, rs61764370 may represent a biologically relevant modifier of tumor behavior whose effects are subtle, context-dependent, and insufficient alone to override established histopathological risk factors.

Future research should prioritize multicenter, prospectively collected cohorts with comprehensive multivariate adjustments, incorporating integrated molecular–histopathological risk models to enhance clinical applicability. This approach is reinforced by recent findings from a Romanian cohort showing a significant association between the GAS5 rs145204276 ins/del polymorphism and colorectal cancer risk—particularly among patients with distal colon tumors, advanced stages, and poorly differentiated histology [46]. These results highlight how regional and histopathological factors may modulate genetic associations, thereby underscoring the value of combining molecular data with tumor-specific pathological features in multicenter studies.

## 5. Conclusions

In this study of patients with histologically confirmed colorectal adenocarcinoma, the rs61764370 TG genotype was not significantly associated with unfavorable histopathological features. Nevertheless, TG carriers showed a tendency toward lower-grade tumors and reduced histopathological aggressiveness compared with wild-type TT carriers. Although these observations do not support a definitive protective effect, they may reflect underlying biological differences, potentially related to altered microRNA binding and modulation of *KRAS* signaling pathways, which could influence tumor differentiation and progression. These partial findings should be interpreted cautiously given the limited sample size, and they should therefore be interpreted as exploratory observations that may provide preliminary insight pending confirmation in larger, independent cohorts to clarify the biological and clinical relevance of the rs61764370 polymorphism in colorectal cancer.

## Figures and Tables

**Table 1:** Baseline characteristics of patients included in study.

Variable	Colorectal Cancer
Number of cases	83
Median age at diagnostic	68 (Min = 45–Max = 87)
Gender	
F	36 (43.4%)
M	47 (56.6%)
Location	
colon	46 (55.4%)
rect	37 (44.6%)
Tumor stage at diagnostic	
I	3 (3.6%)
II	21 (25.3%)
III	39 (47%)
IIV	20 (24.1%)
Differentiation Grade	
Grade 1	28 (33.7%)
Grade 2	42 (50.6%)
Grade 3	13 (15.75)
Perineural invasion	
present	39 (47%)
absent	44 (53%)
Lymphovascular invasion	
present	35 (42.2%)
absent	48 (57.8%)

**Table 2:** Tumor grade distribution according to rs61764370 genotype.

Tumor Grade	Homozygous TT	Heterozygous TG	*p*-Value
1	19 (29.7%)	9 (47.4%)	0.06
2	32 (50.0%)	10 (52.6%)	
3	13 (20.3%)	0 (0.0%)	

## Data Availability

The original contributions presented in this study are included in the article material. Further inquiries can be directed at the corresponding authors.

## Abbreviations

The following abbreviations are used in this manuscript:

CRC	Colorectal cancer
GLOBOCAN	Global Cancer Observatory
TNM	Tumor, Node, Metastasis
LVI	Lymphovascular invasion
PNI	Perineural invasion
3′UTR	3′ untranslated region
LCS6	let-7 microRNA binding site
SNP	Single-Nucleotide Polymorphisms
KRAS	Kirsten rat sarcoma virus
MAPK	Mitogen-activated protein kinase
RNA	Ribonucleic acid
DNA	Deoxyribonucleic acid
MSI	Microsatellite Instability
EGFR	Epidermal growth factor receptor
